# Analysis of the Osseointegration Process of Dental Implants by Electron Paramagnetic Resonance: An In Vivo Study

**DOI:** 10.3390/dj10020028

**Published:** 2022-02-16

**Authors:** Elena Kalinnikova, Margarita Sadovnikova, Alexander Rodionov, Fadis Murzakhanov, Peter Grishin

**Affiliations:** 1 Institute of Doctors Advanced Training of the Chuvashia Health Ministry, 27 Mikhail Sespel Str., 428018 Cheboksary, Russia; elena-vilkova@inbox.ru; 2 Institute of Physics, Kazan Federal University, 18 Kremlevskaya Str., 420008 Kazan, Russia; margaritaasadov@gmail.com (M.S.); rodionovshurik@yandex.ru (A.R.); 3 Dentistry Faculty, Kazan State Medical University, 49 Butlerova Str., 420012 Kazan, Russia; phlus8@mail.ru

**Keywords:** electron paramagnetic resonance, free radicals, osseointegration, surface, dental implants

## Abstract

This research work presents an analysis of the process of an implant’s osseointegration to the jawbone tissue. The purpose of this work was to describe the processes of assimilation and the biochemical dynamics which occur during dental implantation using implants with different macro-microstructure surfaces at the level of stable free radicals using the electron paramagnetic resonance (EPR) method. The experimental investigation was conducted on seven Vietnamese minipigs over twelve months old and weighing up to 30 kg using implants with various macro-microstructure surfaces (SLA, RBM, and HST^TM^) and implantation systems, namely the Adin, Sunran, Biomed, and Osstem systems. The integration of the implant into the bone triggered biochemical processes with the formation of stable free radicals. The EPR method was used to identify the formed paramagnetic species and to study the dynamics of the interaction between the surface of the implant and the bone after one and two months. The concentration of carbonate surface centers increased with the time that the implant was connected to the hard tissue. The “Sunran” and “HST^TM^” were established as the most suitable implantation system and surface type, respectively, thanks to the highest rate of osseointegration (assimilation) with the bone (hard) tissue. Thus, the EPR method provides the opportunity to study implantation processes.

## 1. Introduction

The essential growth of dental implantation procedures has led to interest in the study the mechanisms behind the integration of implants in bone tissues [1,2,3]. The most effective way of assimilating implants is considered to be osseointegration. Osseointegration was first introduced by Professor Per-Ingvar Branemark [4] as the immediate structural and functional coupling of the bone and the surface of a supporting implant [5,6,7,8]. The capacity of an implant to connect with the environmental bone is another basic demand for continuous orthopedic implants. Unsatisfactory osseointegration leads to the formation of fibrous tissues and the subsequent loosening of the prosthesis [9]. The degree of stability (biocompatibility and rejection) achieved during dental implantation and osseointegration depends on the morphological features of the jaw bone or implant (i.e., type of surface roughness, mineral phase density, chemical composition, etc.) [10,11,12,13]. One of the main reasons for osseointegration-related complications are differences between physico-chemical and structural properties of the injured bone tissue and those of the implant. This has already been confirmed by clinical and experimental studies [14,15,16].

Despite the relatively high number of experimental and clinical investigations on this topic (histological [17], biochemical [18], and X-ray analyses [19]), it is currently impossible to make a definite conclusion regarding the kinetics or dynamics of the osseointegration of an implant with the jawbone structure. In our opinion, to remove this blind spot, it is necessary to involve a combination of proven analytical methods to characterize the bone tissue structure after implantation. It is especially important to develop methods to study healing stages through observation of the organic and mineral components of bone tissue at the nano-mesoscopic spatial level [20,21].

The experimental method used for the study of biological materials as biogenic or synthetic in nature is the electron (spin) paramagnetic resonance (EPR or ESR) method [22,23,24,25,26,27,28]. The high sensitivity and spectral resolution, along with the enhanced stability, of current EPR techniques has opened up new opportunities in the study of samples in the last ten years, such as in situ EPR imaging (EPRI) [29]. Traditional EPR can be used to control the impurity of samples or detect the presence of metal impurities [22,30,31]. The EPR method was successfully used to study the assimilation processes between tooth enamel and implant material (paramagnetic native and carbonate radicals with different natures localized in various bone tissue subsystems) and revealed the suitability of the present approach [32]. EPR tomography or EPR-computed tomography (EPR-CT, 3D image) is widely used to study tooth enamel. EPR-CT is used to determine the sites of oxidative stress caused by free radicals. EPR-CT imaging is a powerful method for preferentially revealing free radicals and tracking free radical chemical reactions in vivo [33]. Nevertheless, there are limitations in EPR-CT biological experiments in terms of sensitivity, the data collection rate, the short relaxation time, a lack of perfect reliable spin probes, and sluggish data collection. Free radicals are unbalanced ions with incomplete electron shells that can cause functional changes in molecules, leading to cell damage [34]. The spectral-spatial mode of EPR visualization allows one to register the full spectrum of EPR for each pixel of the image. Using two-dimensional spatial EPR imaging, Philippe Levêque et al. distinguished the dissemination of radicals in stomatological polymer depending on the kind of polymer used [35]. The processes of assimilation with respect to implants and the bone tissue of the jaw have not been studied sufficiently using the EPR approach.

A conventional method for investigating EPR-silent materials involves the formation of specific types of centers by using X-, β-, or γ-rays, or ultraviolet light, and the study of corresponding spectroscopic characteristics. Radiation-induced centers located at phosphate or hydroxyl positions have been revealed in hydroxyapatite, depending on the origin of the synthesis route as well as the irradiation mode [36,37]. Hydroxyapatite (HA) is the mineral component of bone tissue, and synthetic HA has been shown to possess favorable osteoconductive and adhesion properties and has been used as a coating for bone implants [38]. The content of hydroxyapatite in bone tissue can reach 55–75% in terms of weight. Oxygen radicals $O^{-}$, captured hydrogen atoms, holes trapped on ${\mathrm{OH}}^{-}$ and ${\mathrm{PO}}_{4}^{2-}$, the carbonate centers ${\mathrm{CO}}_{2}^{-}$, ${\mathrm{CO}}_{3}^{-}$, ${\mathrm{CO}}_{3}^{3-}$, and color species have been recorded. Coal radicals have been revealed in bones, [39,40]. The spectroscopic and dynamic characteristics of paramagnetic ions can be utilized to determine the radiation dosage in dental enamel [39] and to observe both calcification processes [41] and the co-doping of synthetic samples with diverse ions [24,25]. The impact of various amino acids on the local structure of calcium-deficient hydroxyapatite was also confirmed by using EPR [42]. However, it is worth noting that there is currently a lack of clear information about how both mechanisms and the features of the surface of the implant influence bone assimilation processes studied by the EPR method.

Since the EPR method is susceptible to detecting free radicals and has been proven to be useful in the study of biological objects, the authors of this work suggest the applicability of the EPR approach to the investigation of osseointegration processes. The research was prompted by the following hypotheses: first, that the processes of assimilation (osseointegration) between living bone tissue and the implant’s surface leads to the formation of stable free radicals (spin labels for the EPR technique [30,31]) under biochemical processes; and second, and most importantly, that the EPR method can track the dynamics of the formation of paramagnetic centers depending on the type of implant surface (morphology) and implantation system. Therefore, the purpose of this work is to describe the osseointegration (assimilation) processes and biochemical dynamics which take place during dental implantation using implants with different macro-microstructure surfaces at the level of stable free radicals by using the EPR method. The obtained EPR results will help to establish the most suitable implantation system and an innovative surface with a specific morphology that has osteoregenerative properties.

## 2. Materials and Methods

### 2.1. Investigated Materials

The experimental investigation was carried out on seven Vietnamese minipigs aged over twelve months and weighing up to 30 kg. The morphofunctional characteristics of this breed allow them to be used for conducting dental experiments. The incisors and premolars on both the upper and lower jaws of experimental animals were removed under anesthesia, after which intraosseous implants with various macro-structure coatings (SLA, RBM and ${\mathrm{HST}}^{\mathrm{TM}}$) were installed in the hole of the removed tooth by using the Adin, Sunran, Biomed, and Osstem implantation systems. A solution of thiopental sodium (5%) was used as anesthesia and applied intravenously (10 mL (0.5 g)). The structure of the alveolar processes of the minipigs’ jaws made it possible to install implants of various lengths (8, 10, 12 mm) and with cylindrical diameters of 3.5 mm and 4.5 mm, depending on the thickness of the ridge. After each implant was installed, temporary crowns of self-hardening plastic were made directly in the oral cavity. The stability of the intraosseous implants was determined at different stages during the experiment by using “Osstell mentor” and “Periotest” devices, as well as the X-ray method, and the degree of their osseointegration was assessed [43]. After appropriate exposure in 12% neutral formalin and four-month decalcification in 10% trilon solution, tissue samples were obtained via ascending alcohols, poured into paraffin wax, and both transverse and sagittal sections were prepared with a thickness of 6–8 microns. In this work, three types of surfaces and four implantation systems were studied, which lead to twelve combinations. Six implants were selected for each combination and were installed in slightly different places (to implement randomization and experiment statistics) in the pigs’ jaws. In total, 72 implants were examined using the EPR method.

The SLA surface (sand blasted, large grit, acid-etched) was first developed by Straumann Holding (Basel, Switzerland) and to this day is the most acceptable and widespread technology for the surface treatment of dental implants. The idea behind an SLA-type surface is to improve the biomechanical interaction between the bone tissue and the implant surface by increasing its micro-roughness. The chemical modification of the surface obtained as an outcome of processing also affects the biochemical coupling of the implant with hard tissue. The SLA surface is created via blasting with aluminum oxide followed by the double acid etching of the implants. Several main advantages of the SLA surface have been established, such as a well-developed porous structure with craters that are 2–5 microns in diameter and play a role in the processes of osseointegration. The idea behind the RBM (resorbable blast media) surface to improve the biomechanical coupling between the implant surface and hard tissue by increasing the micro-roughness of the surface. The biomechanical coupling of the implant’s surface and bone is enhanced by calcium phosphate impregnation (CaP) during jet treatment. The “CaP” impregnation allows for the biomineralization of the surface via ion detection and the direct stimulation of cells. The RBM surface (GDT Implants Company Profile, Ashdod, Israel) is created by blasting implants with abrasive calcium phosphate (tricalcium phosphate or hydroxyapatite) and subsequent washing in weak acids. As a result of studying this technology, it was revealed that the RBM surface is structured using a biocompatible medium (abrasive calcium phosphate perceived by the bone), which is completely removed by dissolving in weak acids. Micrographs of the implant surface taken with a scanning electron microscope [44] demonstrate the chaotic structure and the absence of organized micropores on surfaces treated using the RBM method. Humana Dental Implants Accessories Gmbh (Frankfurt am Main, Germany) has developed and implemented ${\mathrm{HST}}^{\mathrm{TM}}$ (Hybrid Surface Treatment technology), a new technological process for the surface treatment of implants. This concept is based on a unique method of surface treatment for Clean & Porous implants, proposed and tested by the world’s leading company specializing in the field of metal surface treatment, Finish Line ltd. As with RBM technology, the HST surface is formed by blasting implants with abrasive calcium phosphate. Unlike the SLA method, the subsequent washing is carried out in weak acids, which eliminates the impact on titanium. Special surface treatment allows for the creation of a highly structured surface with the necessary pores with a diameter of 2–5 microns. The HST surface combines the advantages of the SLA and RBM surfaces described above (highly developed roughness, porosity, and the absence of the foreign elements of the initial alloy—Ti, Al, V). At the same time, the surface of HST is free from the inherent disadvantages of those methods (the incomplete removal of abrasive particles (in the case of SLA) and the insufficiently clear structure of the surface topography (in the case of RBM)). A description of the implantation systems used (Adin, Sunran, Biomed, and Osstem) is beyond the scope of this study and is described in detail in [43].

**Figure 1:**
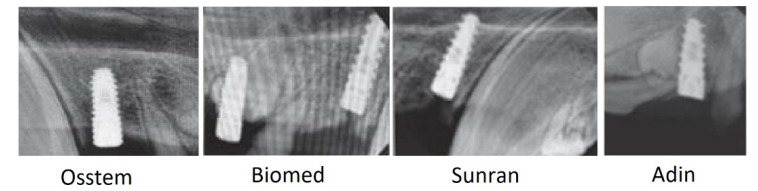
X-ray sampling of targeted radiography of the jaw bones of minipigs with different implantation systems one month after the installation of implants.

**Figure 2:**
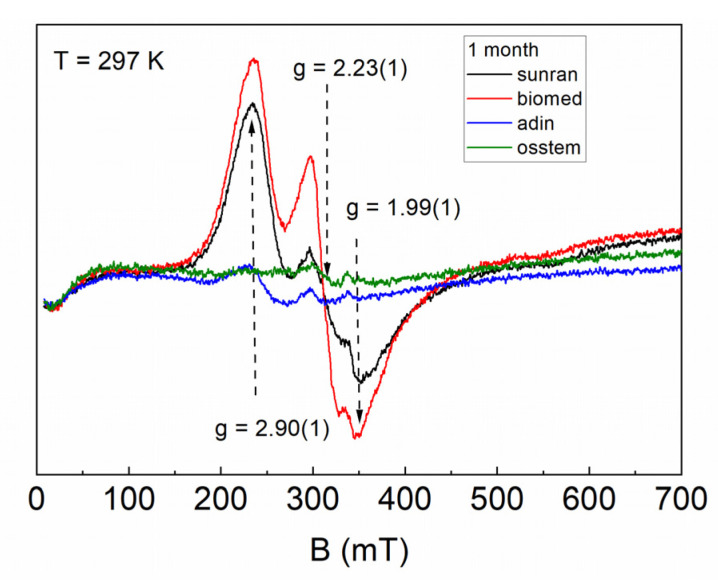
EPR spectra of bone tissue samples before irradiation depending on the implantation system after one month of installation.

The jawbone samples of a Vietnamese minipig (a mineral part consisting of hydroxyapatite) were obtained after 1 month (Figure 1) and 2 months from the start of the experiment by extracting an implant with little bone pieces of pre-implant materials attached to it. The studied Control (raw) sample was the extracted bone tissue obtained before installing the implant and was used for comparative analysis. The appropriate preparation of the jawbone material for EPR study was undertaken by separating it from the implant and sawing it into isolated blocks. All relevant ethical standards and requirements for interventional studies concerning animals, as provided by the Local Ethics Committee of the Federal State Budgetary Educational Institution “Kazan State Medical University” of the Ministry of Health of the Russian Federation (Official Register of the Office for the Promotion of Research on Human Problems registration number 10, dated 22 December 2020, IRB00009490 IORG0007903), were strictly followed.

### 2.2. Research Methods

#### 2.2.1. Theoretical Background

The EPR method is based on resonant (selective) absorption of microwave energy by paramagnetic centers (PC). PCs are atoms, ions, or free radicals (the case for our article) that have their magnetic moment due to the spin (S) or orbital (L) contribution. The main condition of EPR absorption is determined by the expression [30]:

(1) $$h\nu ={g\beta B}_{0}$$

where h is Planck’s constant, ν is the microwave frequency, β is the Boron magneton, g is the spectroscopic splitting factor, and B_0_ is the value of the induction of the magnetic field. The magnetic field is necessary to cause the quantization of PCs along the B_0_ force lines. Thanks to the Zeeman effect, a paramagnetic free radical will have two energy levels, M_S_ = ±1/2, with the energy splitting of magnetic levels equal to ΔE = gβB_0_. Microwave energy, hν, will be absorbed at the transition from a ground state, M_S_ = −1/2, to an excited one, M_S_ = +1/2, if the condition (1) is met. Resonance absorption of the PCs will lead to the appearance of an EPR signal. The intensity (concentration), width, or shape of the EPR line (mechanisms of spin–lattice and spin–spin interactions), and the value of the g-factor (type of paramagnetism) play a critical role in the analysis of free radicals in the material. The gradients of the electric field of the crystal structure lead to the anisotropy of the g-factor, with perpendicular g_⊥_ and parallel g_//_ orientations concerning the vectors B_0_. Thus, these spectroscopic parameters are extremely important for revealing the nature (origin) and studying the behavior of free radicals (paramagnetic centers) in various types of samples. All these spectroscopic values will be discussed in the text of the article [31]. For a detailed description and quantum mechanical interpretation of the method, see articles [30,31].

#### 2.2.2. Experimental Setup

Continuous wave (CW) EPR experiments were performed using the Bruker ESP 300 technique in the X-band (ν_mw_ = 9.6 GHz) frequency range with a magnetic sweep field from 5 mT to 1.5 T. The experimental configurations (modulation A = 0.015 mT, time of integration τ_int_ = 82 ms, and power of mw-source P_mw_ = 150 μW) were tuned to prevent the saturation or distortion of EPR signals. Low-temperature (T < 297 K) experiments were carried out using a flow helium cryostat and a temperature controller. A dialectical ring resonator (ER 4118X-MD5, Bruker BioSpin GmbH, Ettlingen, Germany) was used to register the EPR signal, which significantly increases the sensitivity of the measurements. A cavity with a sample holder allowed for the study of samples up to 3 cm in length and 5 mm wide.

To generate reliable paramagnetic species in the samples under study, X-ray irradiation of the materials was supported by a URS-55 source (U = 50 kV, I = 15 mA, W anticathode) at 297 K for 1 h with a calculated dose of 15 kGy.

The calculation of the measurement error (statistical error) was carried out using the Student’s t-distribution approach. Experiments for each type of surface and implantation system were carried out six times with different samples that were installed and removed independently of each other. This number of experiments made it possible to calculate a confidence interval (error limits) of the order of less than 7% of the measured spectroscopic values with a confidence of 98%.

## 3. Results

Initially, the EPR method was used to study bone tissue samples from the surface of implants installed using various implantation systems. Figure 2 shows the shape (EPR line form) and intensity (peak amplitude) of the signals depending on the implantation method. Since it is known that pure hydroxyapatite is paramagnetically silent, the observed signals can be attributed to native impurity centers in soft tissues and the organic components of bone tissue. The visible distinction in the EPR line was conditioned by the inhomogeneity of the materials under study with different ratios of soft tissues, organic and inorganic phases. The broad intense lines in Figure 2 can be described using three values of g-factors, which refer to an anisotropic spin system with rhombically distorted symmetry. According to the established values, g = 1.99(1), 2.23(1), and 2.90(1), it can be assumed that the resonance lines are associated with complexes of transition metals (Mn, Fe, Cu) which were broadened due to strong spin–spin coupling or interaction with paramagnetic oxygen [22,23].

**Figure 3:**
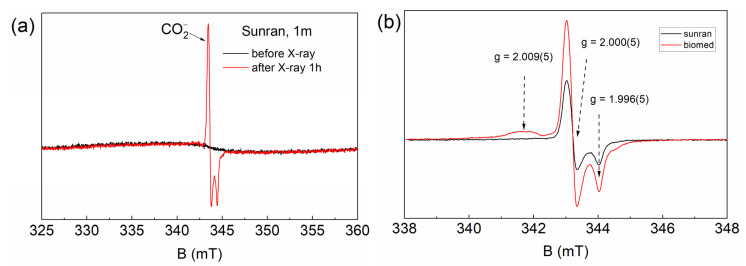
(**a**) Comparison of the EPR lines of the minipig jawbone material after 1 h of irradiation (red) and before irradiation (black). (**b**) The central section of the EPR spectra of bone tissue around the Sunran (black) and Biomed (red) implants after X-ray irradiation.

The EPR spectrum intensity of the resonance absorption is redistributed, indicating the presence of three different impurity centers. The presence of metal ions can be caused by both a small admixture of the implant material (particles of cutting tools) or complexes of iron-containing proteins in the tissues of the studied samples. The described signals are not observed for the control sample (the bone tissue obtained before installing the implant). The procedure for preparing the samples was carried out in a similar way each time, and thus it can be assumed that the signals in Figure 2 and Figure 3 mainly correspond to the implant material. The line width for all EPR spectra is due to the inhomogeneous type of broadening, which was due to the presence of dipole–dipole interaction with the surrounding defects.

Furthermore, to investigate the presence of free radical pre-centers, bone tissue samples were exposed to X-ray irradiation. As an example, Figure 3b shows that the EPR spectrum reflects the content of radiation-induced centers of bone tissue from the different implantation systems. The axial spectroscopic value of g-factors (g = 2.000(5) and g = 1.996(5)) makes it possible to identify the EPR signal as carbonate-based free radicals ${\mathrm{CO}}_{2}^{-}$ type. Previously, applying double resonance techniques with quantum chemical calculations, it was found that carbonate free radical centers occupy the ${\mathrm{PO}}_{4}^{3-}$ position in the structure of hydroxyapatite. It is important to note that the inclusion of carbonate groups in apatite compositions “loosens” the crystal structure of bone tissue, increasing its solubility [30].

**Figure 4:**
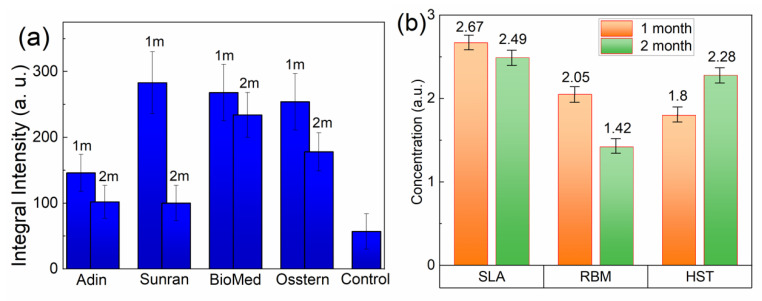
(**a**) Integral EPR intensities of carbonate paramagnetic centers in bone tissue samples adjacent to implants after 1 (1 m) and 2 (2 m) months compared to the control material; (**b**) Comparison of the EPR integral intensities for different types of surface microstructures of the Sunran implant after 1 (orange) and 2 (green) months after implant installation.

The X-ray irradiation procedure was performed for all types of implants with different surface morphologies depending on the time of implant–bone contact (Figure 4a). The integral intensity of the resonant absorption of carbonate radicals strongly depended on the type of implantation system and decreased with the assimilation time. The amplitude of the EPR line depended on the number of impurity paramagnetic ions (free radicals) within the sample. Thus, this method should be used to measure the amount of free (paramagnetic) radicals and other dynamics over time. The integral intensity was obtained via mathematical processing after the double integration of the EPR spectra, since this value allows us to correctly compare concentrations between different samples. Given all the normalizations by sample weight, a comparative analysis of the concentration of free radicals and its change over time for different types of surfaces and implantation systems is shown in Figure 4.

## 4. Discussion

Initially, paramagnetic centers were absent in pure hydroxyapatite, hence no EPR signals were observed in the materials. The presence of impure metal ions (Mn, Fe, Cu, etc.) in the crystal structure of the studied samples or the procedure of X-ray irradiation caused the formation of irradiation centers, which were effectively detected using EPR spectroscopy. The powder spectra of carbonate radicals in implants (radiation-induced centers) were characterized by a spin Hamiltonian of axial symmetry:

H = g_//_βB_z_S_z_ + g_⊥_β(B_x_S_x_ + B_y_S_y_) (2)

where g_//_ and g_⊥_ are the principal components of the g tensor, B_i_, S_i_, and I_i_ are the projections of the strength of the external magnetic field and the electron (S = 1/2) spin onto the i = {x, y, z} coordinate axis, and β is the Bohr magneton. In the samples studied, there were no magnetic nuclei in free radicals (^13^C, I = 1/2, 1.4% abundant), so we do not take into account the contributions from electron-nuclear interactions to the Hamiltonian (the state operator of the spin system). In EPR spectroscopy, g-factor parameters play an important role, since these values reflect local symmetry and are responsible for the nature of the paramagnetism of the ion under study. The presence of axiality indicates the presence of electric floor gradients in the lattice structure. We do not give an extended spin Hamiltonian for transition group metal ions since in this paper they are registered as broadened structureless lines.

The spin Hamiltonian parameters (2) and the shape of the EPR line for the identical paramagnetic center can vary significantly depending on the kind of sample or surface. This may be related to various atomic environments, the level of deformation in the crystal lattice, or the degree of delocalization with respect to the paramagnetic center [24,25]. The differences in the parameters of the spin Hamiltonian are useful for subsequent analysis of the crystal structures of samples doped with various cations [30,31].

The most widespread and studied radiation-induced signals relate to carbonate radicals [45,46]. This kind of carbonate radical (point defect) has an electron spin S = 1/2 and an anisotropic form of the resonance line with the g-factor of about 1.99–2.01 (g_//_ > g_⊥_). Such a change in the g-factors was due to the anisotropy of the crystal field being dependent on the position of the impurity center and serves as a source of additional information about the localization of the radical in the sample lattice. In the material under study, the carbonate anions function as spin traps and can capture electrons when exposed to X-ray or gamma radiation, due to which impurities acquire paramagnetic properties.

The noticeable decrease of carbonate radicals in the second month of the experiment was a result of active collagen-containing unpaired electrons penetrating the pores of hydroxyapatite, forming stable chemical bonds with the surface of the material. A comparison of the integral EPR intensities depending on the time the implant was kept in the bone tissue showed that the data for the Sunran implantation system underwent the greatest changes (Figure 4a), tending towards low values in Control sample, and this correlates qualitatively with previous results [32]. Carbonate radiation-induced centers are extremely stable (even at elevated temperatures) and can be used successfully as spin probes to study the local environment [47]. The origin of these centers is associated with bio oxidative processes occurring in bone tissue (between mineral hydroxyapatite and collagen) during the formation of an organo-mineral phase. It is worth noting that a prerequisite for the application of this method is the presence of paramagnetic centers, which is a limitation of the experimental method. Considering the obtained EPR data, the Sunran implant had the highest rate of osseointegration and is more biocompatible with bone tissue. With respect to the Biomed and Osstem implantation systems, the assimilation rate was lower but was also statistically significant. Previous experimental studies (histological [17] and biochemical [18] and X-ray analyses [19]) have also shown how the degree of osseointegration depends on various methods of implantation systems.

Since “Sunran” was established as the most suitable implantation system, it was necessary to find out which of the innovative surfaces with specific morphologies stimulates osteoregenerative properties. To study the surface features, the EPR spectra of carbonate centers in the mineralized part of the bone tissue were analyzed. A similar pattern was revealed, comparing the EPR spectra and the concentration of free radicals in bone tissue after two months from the start of the experiment (Figure 4b). The process of mineral-organic matter formation lead to the formation of pre-centers of carbonate radicals, which are typically present in bone tissue. Figure 4b shows that for three types of surfaces (SLA, RBM, and HST^TM^), the integral intensity of the EPR spectra decreases with time, indicating the slow mineralization of bone tissue. The reverse condition is observed for the HST^TM^ surface. The concentration of carbonate surface centers increases over time when the implant interacts with the bone. The obtained result prompts the unambiguous conclusion that the most preferred surface for assimilation processes is the HST^TM^ material. However, it does not follow that the other types of surfaces are unsuitable for bone engineering, as hard tissue mineralization processes are highly complex and require more detailed study.

## 5. Conclusions

We have described the processes of osseointegration (assimilation) and biochemical dynamics during dental implantation using implants with different macro-microstructure surfaces at the level of stable free radicals using the EPR method. The concentration of carbonate surface centers increases when the implant interacts with the bone, since the integration of the implant into the bone causes biochemical processes with the formation of stable free radicals. The EPR method was used to identify the formed paramagnetic centers and to study the dynamics of the interaction between implant surface and the bone. The origin of these centers is associated with bio oxidative processes occurring in bone tissue (between mineral hydroxyapatite and collagen) during the formation of an organo-mineral phase.

The noticeable decrease of carbonate radicals in the second month of the experiment was due to active collagen-containing unpaired electrons penetrating the pores of hydroxyapatite, forming stable chemical bonds with the surface of the material. The “Sunran” and “HST^TM^” were established as the most suitable implantation system and surface type, respectively, thanks to the highest rate of osseointegration (assimilation) with bone (hard) tissue. The obtained EPR results enables the most suitable implantation system and type of innovative surface with a specific morphology for stimulating osteoregenerative properties to be determined. Thus, through its high sensitivity and spectral resolution, the EPR method provides the opportunity to study implantation processes.

## Data Availability

Data can be available upon request from the authors.
